# Role in Preventing Alcoholic Liver Disease Progression: A Comparative Study of Whole-Component Finger Citron Essential Oil and Its Major Component D-Limonene

**DOI:** 10.3390/nu17071255

**Published:** 2025-04-03

**Authors:** Jingxin Chen, Genghua Ou, Wenting Gu, Jian Shi, Ruiying Lyu, Xueping Wu, Junming Wang, Chunhong Liu

**Affiliations:** 1 College of Food Science, South China Agricultural University, Guangzhou 510642, China; 20222145004@stu.scau.edu.cn (J.C.); 13600091590@163.com (G.O.); gwtgz2020@126.com (W.G.); clinicalshijian@foxmail.com (J.S.); 20223185044@stu.scau.edu.cn (R.L.); xp158165@163.com (X.W.); 20223141071@stu.scau.edu.cn (J.W.); 2 Guangdong Provincial Key Laboratory of Food Quality and Safety, Guangzhou 510642, China

**Keywords:** alcohol-induced liver injury, finger citron essential oil, hepatoprotection, antioxidation, anti-inflammation

## Abstract

**Background/Objectives**: Chronic alcohol overconsumption triggers alcohol liver injury, and therapeutic strategies targeting alcohol-triggered oxidative stress and hepatic inflammatory responses represent potential approaches to ameliorating alcohol-related hepatotoxicity. This study aimed to determine the hepatoprotective activity of finger citron essential oil (FCEO) in alcoholic liver disease (ALD)-afflicted rats and explore its underlying mechanisms. In order to identify the effective components, we compared the effects of FCEO and D-limonene. **Methods**: The regulatory effects of FCEO on metabolic enzymes were systematically evaluated through in vitro experiments. In vivo studies were conducted to investigate and compare the hepatoprotective effects of FCEO and D-limonene. Staining methods, assay kits, and Western Blot were used to determine the roles of FCEO and D-limonene in the ALD rats. **Results**: We found that FCEO downregulated phase I metabolic enzymes and upregulated phase II metabolic enzymes in Buffalo Rat Liver-3A (BRL-3A) cells. FCEO and/or D-limonene intervention reduced transaminase levels in ALD rats and effectively alleviated inflammatory cell infiltration and lipid droplet accumulation in their liver tissue. Additionally, FCEO and D-limonene played a regulatory role in oxidative stress and inflammation-related pathways such as the MAPK/Nrf2 and NF-κB/AMPK pathways. FCEO was superior to D-limonene as an antioxidant in alleviating alcoholic liver injury. **Conclusions**: This study revealed the alleviative effects and mechanisms of FCEO on alcoholic liver injury, demonstrating better efficacy compared to its monomer, thus providing a strategy for the development and utilization of finger citron resources.

## 1. Introduction

Alcoholic liver disease (ALD) encompasses a continuum of hepatic metabolic disorders, progressing through reversible stages (an alcoholic fatty liver, alcoholic hepatitis) and advancing to irreversible pathological states, including liver fibrosis and cirrhosis, ultimately resulting in hepatocellular carcinoma or liver failure [1].

The pathogenesis of ALD is intricate, encompassing the direct and indirect toxic effects of ethanol, the generation and accumulation of reactive oxygen species (ROS), cell injury, steatosis, oxidative stress, inflammatory cascade responses [2], intestinal barrier dysfunction [3], dysbiosis [4], and hepatotoxicity from toxic metabolites [5].

Ethanol metabolism disrupts the equilibrium between lipid anabolism and lipolysis, leading to hepatic triglyceride (TG) and total cholesterol (TC) accumulation, precipitating the initial manifestations of ALD, specifically alcoholic fatty liver [6]. It also enhances intestinal permeability, promoting liver macrophage activation to generate substantial quantities of ROS, pro-inflammatory cytokines, and adhesion molecules, all of which contribute to liver injury [7]. Oxidative stress constitutes the primary pathogenic trigger in ethanol-induced hepatotoxicity, with mitochondrial ROS overproduction mediated by cytochrome P450 2E1 (CYP2E1) serving as the initiating molecular event following chronic alcohol exposure [8]. Central to antioxidant defense, nuclear-factor-erythroid-2-related factor 2 (Nrf2) mitigates ethanol-induced oxidative damage through transcriptional activation of phase II detoxification enzymes, particularly heme oxygenase-1 (HO-1) and NAD(P)H:quinone oxidoreductase 1 (NQO1) [9,10]. In parallel, chronic alcohol consumption activates pro-inflammatory signaling via nuclear factor-kappa B (NF-κB) pathway induction, activated by endotoxins and cytokines that ultimately drive hepatocyte necrotic death [11]. Mitogen-activated protein kinase (MAPK) signaling plays dual roles in alcohol liver injury, responding to inflammatory signals [12] and regulating Nrf2 [13].

AMP-activated protein kinase (AMPK) and SIRT1 (Sirtuin 1) have been observed to synergize to exert protective effects in murine models of inflammation-related disorders, including ALD [14]. Persistently activated AMPK exerts suppressive effects on NF-κB signaling pathways, thereby attenuating transcriptional upregulation of pro-inflammatory mediators and subsequently ameliorating inflammatory damage [15]. SIRT1 acts directly on NF-κB and downregulates the expression of pro-inflammatory genes [16]. It is evident from the previous research that modulating inflammation and oxidative stress may be a key strategy for treating and understanding alcoholic liver injury. Recent studies have demonstrated the therapeutic potential of medicinal and edible homologous substances in addressing alcoholic liver injury, with bioactive compounds like *Lycium barbarum* polysaccharides exhibiting hepatoprotective effects through antioxidant pathways [17] and puerarin showing anti-inflammatory efficacy [18].

Finger citron is the dried fruit of *Citrus medica* L. var. *sarcodactylis Swingle* (from the family Rutanceae). Finger citron is known for its diverse pharmacological properties, including its antioxidant, anti-inflammatory, and anti-dyspeptic effects. In traditional Chinese medicine, finger citron is employed to cure the recession of Qi in the liver and stomach [19,20]. And its essential oil components have also been reported to have anti-inflammatory [21] and antioxidant activities [22]. The intake of finger citron enhances glycolipid metabolism, liver function, and mitigated liver lipid peroxidation [23]. These bioactive properties are critical for both inhibiting the progression of ALD and facilitating early-stage intervention. Notable herbal medicines such as *Notoginseng radix*, *Cnidii Fructus*, and *Lycii Fructus* have demonstrated therapeutic effects against ALD through multimodal mechanisms encompassing antioxidant activity, anti-inflammatory responses, and hepatic lipid metabolism regulation [24]. Additionally, D-limonene, one of the main components of FCEO, has been reported to act as an effective hepatoprotective agent due to its antioxidant and anti-inflammatory properties [25]. Therefore, we believe that FCEO also has the potential to mitigate alcohol-induced liver injury. With the modernization of traditional Chinese medicine, many drugs are now natural or semi-natural products [26]. The active components of herbal medicines and their pharmacological mechanisms have long been the focus of relevant research. It has also been proposed that there are differences in pharmacological activity between whole plant extracts and individual components [27]. Based on the view that a complex phytochemical composition can promote the biological activity of an extract at multiple targets, as well as the hepatoprotective capabilities of limonene, FCEO may possess an enhanced hepatoprotective potential allowing it to ameliorate alcoholic liver injury through phytochemical synergistic interactions.

To elucidate the therapeutic potential of and underlying mechanisms through which FCEO alleviates alcohol liver injury, we first investigated the effects of FECO on liver metabolism in cellular experiments and then compared the effects of FCEO and D-limonene in alleviating liver injury in ALD rats. This study could provide a theoretical basis for developing functional food and health products harnessing the benefits of FCEO, especially for hepatoprotection.

## 2. Materials and Methods

### 2.1. Materials

Finger citron essential oil was obtained from Zhejiang Jinshoubao Biomedical Technology Co. (Jinhua, China), and d-limonene was obtained from Aladdin (Shanghai, China).

### 2.2. GC-MS Analysis

Qualitative and quantitative analyses of FCEO samples were conducted using a gas chromatography/mass spectrometer (GC-MS) (Thermo Fisher Scientific, Waltham, MA, USA). For GC analysis, 1.0 μL of the sample was diluted with ethanol absolute (1:80 *v*/*v*) and injected into the TG-5 MS (250 μm × 60 m, 0.50 μm) at 250 °C in a split ratio of 1:10. The carrier gas (helium) flow was 1.2 mL/min. The applied temperature gradient was as follows: the initial temperature was 50 °C, which was held for 2 min and then raised by 10 °C/min to 110 °C; subsequently, the temperature was increased by 3 °C/min to 200 °C and then by 10 °C/min to 230 °C and finally maintained at 250 °C for 5 min. For MS analysis, the equipment was operated in electron impact ionization mode at 70 eV; the mass range scanned was 50–550 amu in full-scan acquisition mode; ion source and quadrupole temperatures were set to 280 °C and 150 °C, respectively; and a solvent delay of 12 min was applied. The volatile compounds obtained via the GC-MS were compared with retention indices and spectra from National Institute of Standards and Technology (NIST) Library (matches: > 85%).

### 2.3. Cell Culture and Treatment

The Buffalo Rat Liver-3A (BRL-3A) cell line was obtained from Procell (Procell Life Science and Technology, Wuhan, China). Cultures were propagated in MEM containing 10% fetal bovine serum (FBS, Gibco, Grand Island, NY, USA) and 1% penicillin–streptomycin (Beijing Labgic Technology, Beijing, China) under standard culture conditions (5% CO_2_, 37 °C). A 10% FCEO stock solution was prepared by dissolving 1 mL of FCEO in 9 mL of DMSO. For cell treatment, 1 μL of the stock solution was added per 1 mL complete medium to achieve a 600 μM working concentration. Two-fold serial dilutions with complete medium yielded subsequent concentrations of 300, 150, and 75 μM.

Logarithmic-phase cells were seeded into 96-well plates. Following a 24 h incubation, the culture medium was aspirated, and then 100 μL FCEO solutions were added at concentrations ranging from 0 to 600 μM (75, 150, 300, and 600 μM). After 2 h of FCEO treatment, optimal FCEO concentrations were determined using a CCK-8 viability assay (Beijing Biosynthesis Biotechnology Co., Ltd., Beijing, China).

### 2.4. Animals

Sixty 5-week-old male SD rats (110–120 g) were purchased from the Guangdong Medical Laboratory Animal Center (License No. SYXK (Guangdong) 2022-0002). All animal experimental procedures strictly adhered to the ARRIVE guidelines and were approved by the Institutional Animal Ethics Committee of South China Agricultural University (Permission No. 2022B016). The rats were allowed a seven-day acclimatization period before the formal experiments were conducted. All rats were maintained in an enclosure kept at 22.0 ± 0.5 °C with 50–60% relative humidity under standardized lighting conditions (12:12 light/dark cycle). Rats were given free access to standardized feed and purified water and co-housed in pairs within cages.

### 2.5. Experimental Groups and Treatments

All rats were randomly divided into six groups (n = 10) after 1 week of acclimatization. FCEO solutions were prepared using corn oil (Arawana, Guangzhou, China) and saline, respectively. Edible alcohol was purchased from RED STAR Co., Ltd. (Beijing, China). The rats were subjected to two daily gavage sessions for 7 periods, with 4 days per period, amounting to a total of 28 days (Table 1). The rats underwent twice-daily oral gavage sessions: in the first, the FCEO solution, D-limonene solution, and corn oil were administered at 9 a.m.; in the second, the edible alcohol solution and saline were administered at 2 p.m. The groups were established according to the FCEO or D-limonene gavage concentration administered: the control group (C) was administered pure corn oil and normal saline; the ethanol treatment group (ET) was given pure corn oil and edible alcohol; the FCEO low-dose group (FL) received 100 mg/kg of FCEO and edible alcohol; the FCEO medium-dose group (FM) was administered 200 mg/kg of FCEO and edible alcohol; the FCEO high-dose group (FH) was given 400 mg/kg of FCEO and edible alcohol; and the D-limonene group (LM) was administered 116 mg/kg of D-limonene and edible alcohol, equivalent to the quantity of D-limonene administered in the FCEO medium-dose group. FCEO and D-limonene were administered via gavage at 4 mL/kg body weight, and group C was given 8–10 mL/kg (via gavage) of saline, while the remaining groups were administered (via gavage) edible alcohol with an increasing concentration gradient distilled with saline. In gavage period I, 8 mL/kg of 20% alcohol by volume (ABV) edible alcohol was administered; in gavage period II, 9 mL/kg of 30% ABV edible alcohol was administered; in gavage period III, 10 mL/kg of 40% ABV edible alcohol was given; and in gavage periods IV to VII, 10 mL/kg of 56% ABV edible alcohol was administered. The original edible alcohol (56% ABV) was diluted with saline via a volumetric method to prepare 20%, 30%, and 40% ABV solutions before use. The FCEO administration protocols were based on a standard human adult dose proportional to the surface area of a rat’s body as per the Chinese Pharmacopoeia. The body weights of all rats were monitored in 4-day intervals.

### 2.6. Biochemical Parameter Assays

Rats were fasted for 12 h, and, following anesthesia with sodium pentobarbital, orbital sinus blood collection was performed. Euthanasia for cervical dislocation was carried out prior to systemic dissection, during which liver tissue was weighed before being harvested. The orbital blood was placed in refrigerator at 4 °C overnight, and serum was obtained via centrifugation at 3000× *g* for 15 min before being frozen at −80 °C.

Liver tissue, which was collected in centrifuge tubes, was ground with phosphate-buffered saline buffer (Procell Life Science and Technology, Wuhan, China). Liver tissue homogenates were centrifuged at 12,000× *g* for 10 min at 4 °C. The resultant supernatant was collected for subsequent biochemical analyses.

ALT, AST, TG, and TC levels in serum and TG, TC, and GSH levels in the liver were determined according to kit instructions (Nanjing Jiancheng Bioengineering Institute, Nanjing, China). The levels of IL-1β and TNF-α in serum were analyzed using commercial enzyme-linked immunosorbent assay (ELISA) kits (Shanghai Jiang Lai Biotechnology Co., Ltd., Shanghai, China).

### 2.7. Liver Histopathological Examination

Liver tissues were removed and fixed in 4% paraformaldehyde prior to paraffin embedding; this was followed by transverse sectioning at a thickness of 6 μm. Deparaffinized sections were then rehydrated and subsequently stained with hematoxylin–eosin (HE) and oil red O. We used an upright microscope (Leica DM4 B, Wetzlar, Germany) to make observations and a digital micro-imaging system to capture photographs.

### 2.8. Measurement of Phase I and Phase II Metabolic Enzymes

Logarithmically growing BRL-3A cells exhibiting optimal viability were inoculated into sterile polystyrene 6-well plates. Following 24 h of apposition, experimental groups underwent 2 h of exposure to different FCEO concentrations (0, 75, 150, and 300 μM), while the control group was incubated with 0.1% DMSO. Quantitative analysis of CYP1A2, CYP2E1, CYP3A4, GST, and UGT1 levels in the cells was performed using commercial enzyme-linked immunosorbent assay (ELISA) kits (Shanghai Jiang Lai Biotechnology Co., Ltd., Shanghai, China). BCA was used to measure the total protein in each group using commercially available kits (Beyotime, Shanghai, China).

### 2.9. Protein Extraction and Western Blot

Rat liver tissue was homogenized using RIPA buffer, adding a PMSF, protease, and phosphatase inhibitor cocktail (Beyotime, Shanghai, China) and carrying out grinding in a frozen mixed grinding apparatus to extract total protein. Total proteins were isolated via centrifugation at 12,000× *g* for 10 min at 4 °C. After adding the loading buffer, we adjusted the protein concentrations of all the samples to the same concentration, usually 5 mg/mL, and boiled them for 8 min.

Western blot analyses were performed with the following antibodies: anti-Nrf2 (Abcam, Cambridge, UK, AB137550, 1:1000), anti-NQO1 (Proteintech, Wuhan, China, 11451-1-AP, 1:1000), anti-HO-1 (Cell Signaling Technology, Danvers, MA, USA, 43966S, 1:1000), anti-p-NF-κB (Cell Signaling Technology, 3033S, 1:1000), anti-p-JNK (Cell Signaling Technology, 4671S, 1:1000), anti-JNK (Cell Signaling Technology, 9252S, 1:2000), anti-p-ERK (Cell Signaling Technology, 4370S, 1:1000), anti-ERK (Cell Signaling Technology, 4695S, 1:1000), anti-p-p38 (Cell Signaling Technology, 4511S, 1:1000), anti-p38 (Cell Signaling Technology, 9212S, 1:1000), anti-p-AMPK (Cell Signaling Technology, 2513S, 1:1000), anti-AMPK (Cell Signaling Technology, 2532S, 1:1000), anti-GAPDH (Proteintech, 10494-1-AP, 1:6000), anti-Tublin (Affinity Biosciences, Nanjing, China, AF7011, 1:6000), and anti-β-actin (Abmart, Shanghai, China, M20011, 1:5000) diluted with 5% BSA (Biofroxx, Alzenau, Germany). Protein samples and markers were resolved on SDS-PAGE gels (Epizyme Biomedical Technology, Shanghai, China) under denaturing conditions. After separation via electrophoresis, protein samples were transferred onto polyvinylidene difluoride (PVDF) membranes (0.45 μm, Millipore, Burlington, MA, USA) and shaken lightly in 5% skimmed milk powder solution for blocking. Following primary antibody incubation (4 °C, 16 h), immunoblotted membranes underwent 3 sequential TBST rinses before treatment with HRP-linked secondary antibodies ((Affinity Biosciences) 1:8000 dilution at room temperature for 90 min). After secondary-antibody incubation, chemiluminescent detection was achieved using ECL reagent (BioSharp, Hefei, China). Band intensity was semi-quantitatively analyzed using ImageJ software (https://imagej.net/ij/download.html).

### 2.10. Statistical Analysis

Statistical analyses were performed using IBM SPSS Statistics 25 software. One-way ANOVA was used to confirm whether there were differences between groups. The results of the analyses were expressed as means ± SD. *F*-values (between-group *df* and within-group *df*) reflect the magnitude of differences between two or more groups, with *p* < 0.05 indicating a statistically significant difference and *p* < 0.01 indicating that highly significant differences were accepted between groups. In the statistical analyses, we employed the Least Significant Difference (LSD) test when variance homogeneity was confirmed, whereas the Games–Howell approach was selected under conditions of heteroscedasticity. The final data were plotted using GraphPad Prism 8.0.2 software.

## 3. Results

### 3.1. GC–MS Results

The GC-MS results are shown in Supplementary Figure S1, and the main constituents of FCEO are shown in Table 2.

### 3.2. Influence of FCEO in Cell Viability

**Figure 1:**
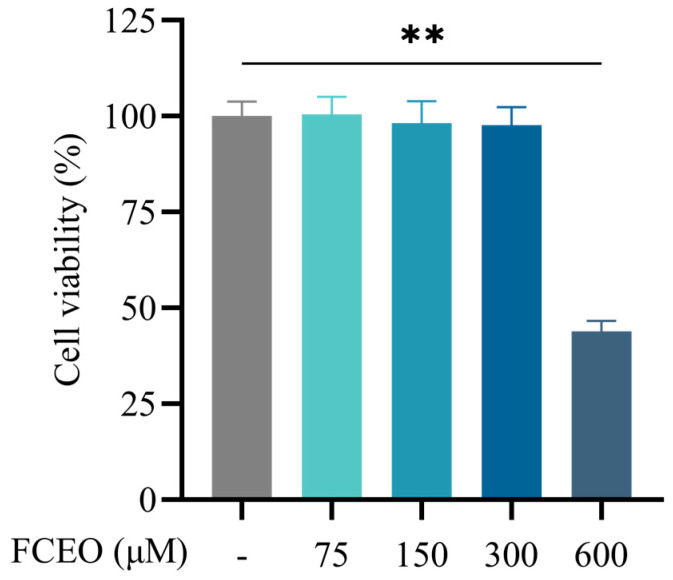
Cell viability of FCEO treatment on BRL-3A cells. Compared to the control group, ** represents *p* < 0.01. Abbreviations: FCEO, finger citron essential oil.

Cell viability was assessed through cck-8 assays following 2 h of treatment with varying concentrations (75, 150, 300, and 600 μM) of FCEO on BRL-3A cells. The results are displayed in Figure 1. Compared with the control group, the concentrations of 75, 150, and 300 μM of FCEO had no significant effect on cell viability, while FCEO at 600 μM significantly reduced cell viability (*F*_4, 25_ = 108.01). Therefore, 75, 150, and 300 μM of FCEO were selected for the following intervention experiments.

### 3.3. Influence of FCEO on Phase I and Phase II Metabolic Enzymes

**Figure 2:**
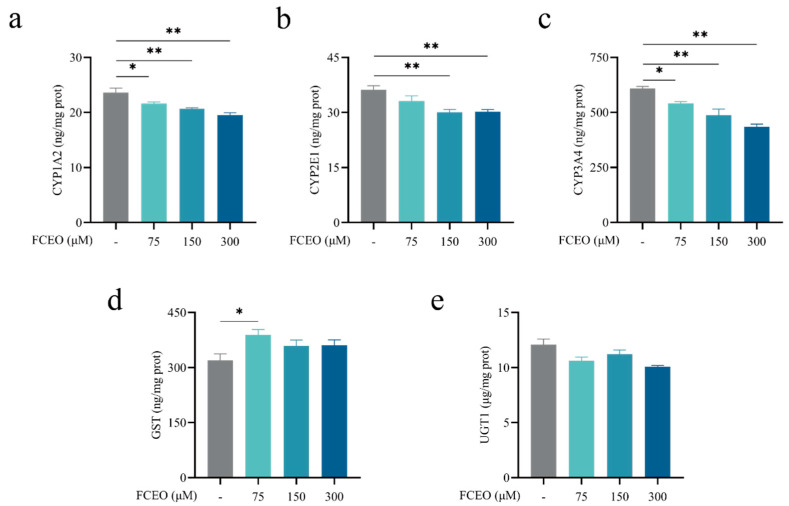
Modulation of phase I and II metabolic enzymes by FCEO in BRL-3A cells: (**a**) CYP1A2 content; (**b**) CYP2E1 content; (**c**) CYP3A4 content; (**d**) GST content; (**e**) UGT1 content. The experimental graphs are expressed as means ± SD (n = 4). Compared to the control group, * represents *p* < 0.05, and ** represents *p* < 0.01. Abbreviations: FCEO, finger citron essential oil; CYP, cytochrome P450 proteins; GST, glutathione S-transferase; UGT, glycoprotein glucosyltransferase.

The effects of FCEO on phase I and II enzymes in BRL-3A cells were analyzed after treatment with 75, 150, and 300 μM concentrations of FCEO for 2 h. The levels of CYP1A2, CYP2E1, and CYP3A4 are shown in Figure 2a–c. There was a dose–effect relationship between FCEO concentration and phase I metabolic enzyme levels, and FCEO significantly decreased CYP1A2 (*F*_3, 12_ = 10.96), CYP2E1 (*F*_3, 12_ = 7.74), and CYP3A4 (*F*_3, 12_ = 19.91) levels compared to the control group. GST and UGT1 are phase II metabolic enzymes. As shown in Figure 2d,e, in the BRL-3A cells under the same treatment conditions, the level of GST was significantly increased by the addition of 75 μM of FCEO, while no significant differences were found in the other concentrations (*F*_3, 12_ = 3.156). Also, FCEO had no significant effect on UGT1 levels (*F*_3, 12_ = 5.42).

### 3.4. Effect of FCEO and D-Limonene Intervention on Weight and Hepatic Index in ALD Rats

**Figure 3:**
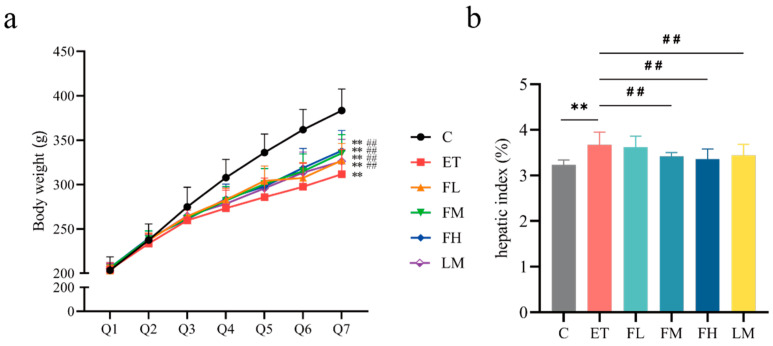
Effects of FCEO or D-Limonene intervention on bodyweight and hepatic index in rats with alcohol-induced injuries. (**a**) Bodyweight changes of rats (n = 10); Q1~Q7 denote gavage period I to gavage period VII, respectively, with every 4 days being a gavage period. (**b**) Hepatic index (n = 10). Compared to the control group, ** represents *p* < 0.01; compared to the ET group, ## indicates *p* < 0.01. Abbreviations: FCEO, finger citron essential oil; C, control group; ET, ethanol treatment group; FL, FCEO low-dose group; FM, FCEO medium-dose group; FH, FCEO high-dose group; LM, D-limonene group.

The changes in the bodyweights of the rats in each group at different intragastric administration periods are shown in Figure 3a. The initial body weights of all the animals in the experimental groups were not significantly different at baseline, and a gradual increase in weight was observed in all the groups throughout the study. By the third period, the bodyweights of the rats in control group were significantly higher than those of the rats in the other experimental groups, and this difference persisted until the end of the study (*F*_5, 54_ = 12.89), indicating that the intake of alcohol might have affected the feeding behavior and energy intake of the rats, resulting in a decrease in bodyweight. Starting in the fifth period, the FCEO intervention rats demonstrated significant weight gain relative to the ET group, with parallel increases observed in the LM group.

The ET group exhibited significantly increased hepatic indices compared to the control group, while the FM, FH, and LM groups showed significant reductions relative to the ET group (Figure 3b) (*F*_5, 54_ = 2.27). These results suggest that the administration of FCEO or D-limonene could alleviate body weight loss and abnormal liver index values caused by alcohol intake.

### 3.5. Histopathological Examination

**Figure 4:**
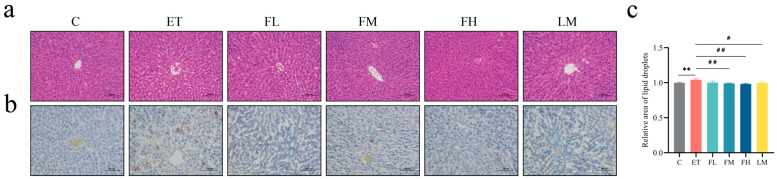
Histopathological observations of rats with alcohol-induced injuries under a light microscope: (**a**) HE stain (200×), (**b**) Oil Red O stain (200×), and (**c**) relative area of lipid droplets (n = 3). Scale: 100 μm. Compared to the control group, ** represents *p* < 0.01; compared to the ET group, # indicates *p* < 0.05, and ## indicates *p* < 0.01. Abbreviations: FCEO, finger citron essential oil; C, control group; ET, ethanol treatment group; FL, FCEO low-dose group; FM, FCEO medium-dose group; FH, FCEO high-dose group; LM, D-limonene group.

The histopathological examination results are shown in Figure 4. As shown through HE staining (Figure 4a), the liver tissue of the ET group was structurally disordered, with blurred hepatocyte boundaries, enlarged cells, irregular fatty vacuoles, and inflammatory cell infiltration. After treatment with different doses of FCEO or D-limonene, the fatty vacuolization of hepatocytes in the rats was reduced, which was conducive to reducing the alcohol-induced structural damage of the hepatocytes. Oil Red O staining showed there was a more positive region in the ET group than in the C group. Additionally, the positive regions in the FM, FH, and LM groups were significantly smaller than those in the ET group (Figure 4b), while there was no significant difference between the LM group and the FCEO intervention groups, indicating that fat deposition was suppressed by treatment with FCEO or D-limonene (*F*_5, 12_ = 6.88).

### 3.6. Effects of FCEO and D-Limonene Intervention on Aminotransferase and Lipometabolism in ALD Rats

**Figure 5:**
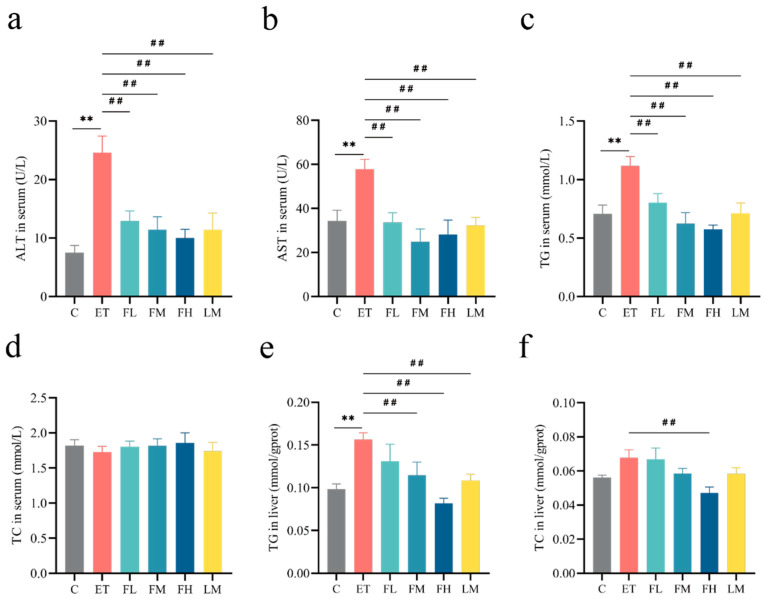
Effects of FCEO or D-limonene intervention on hepatic transaminase activities and lipid parameters in ALD rats. (**a**) ALT in serum (normal reference range = 1.0–223.3 U/L); (**b**) AST in serum (normal reference range = 0.2–838.3 U/L); (**c**) TG in serum (normal reference range = 0.2–2.7 mmol/L); (**d**) TC in serum (normal reference range = 0.4–2.1 mmol/L); (**e**) TG in liver tissue; (**f**) TC in liver tissue (n = 6). Compared to the control group, ** represents *p* < 0.01; compared to the ET group, ## indicates *p* < 0.01. Abbreviations: FCEO, finger citron essential oil; ALD, alcohol-induced liver disease; C, control group; ET, ethanol treatment group; FL, FCEO low-dose group; FM, FCEO medium-dose group; FH, FCEO high-dose group; LM, D-limonene group; ALT, alanine aminotransferase; AST, aspartate aminotransferase; TG, triglyceride; TC, total cholesterol.

Figure 5a,b demonstrate that serum ALT and AST levels, established biomarkers of hepatic damage, exhibited significant increases following alcohol intake but remained within the normal range. Different doses of FCEO or/and D-limonene treatment significantly downregulated ALT (*F*_5, 30_ = 7.86) and AST (*F*_5, 30_ = 5.36).

As shown in Figure 5c,e, both in serum and in liver tissues, alcohol treatment significantly increased the levels of TG (serum TG *F*_5, 30_ = 6.11, liver TG *F*_5, 30_ = 4.97), but no significant effect was found for TC (serum TC *F*_5, 30_ = 0.23, liver TC *F*_5, 30_ = 3.35). Medium and high doses of FCEO and D-limonene restored the levels of TG. At the same time, no significance was observed for TC in any of the groups, except in the high-dose-of-FCEO group relative to the ET group, as shown in Figure 5d,f. These results indicate that FCEO and its main component D-limonene might help to protect rat livers from alcohol-induced injury as well as lipometabolism disorders.

### 3.7. Effects of FCEO and D-Limonene Intervention on Oxidative Stress and MAPK/Nrf2 Pathway in ALD Rats

**Figure 6:**
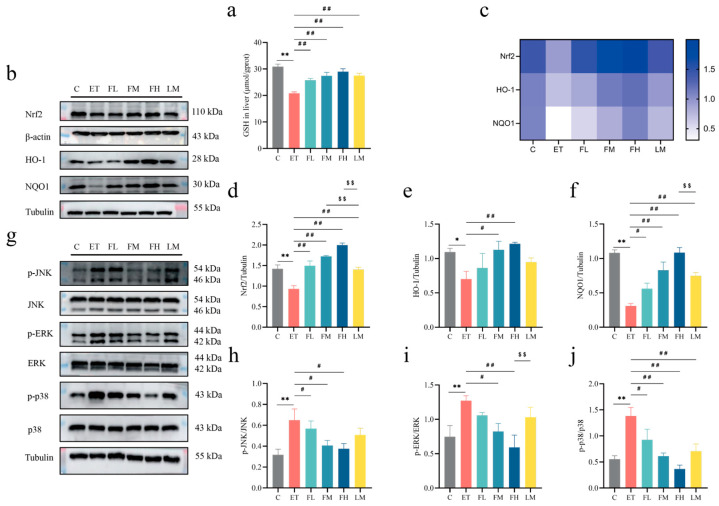
Effects of FCEO and/or D-limonene intervention on antioxidant enzyme and MAPK/Nrf2 pathways in liver tissue of ALD rats: (**a**) liver tissue GSH content (n = 6); (**b**) Western Blot strip map of Nrf2 pathway proteins, (**c**) heat map corresponding to the content of proteins associated with the Nrf2 pathway, (**d**) Nrf2, (**e**) NO-1, (**f**) NQO1, (**g**) Western Blot strip map of MAPK pathway proteins, (**h**) p-JNK/JNK, (**i**) p-ERK/ERK, and (**j**) p-p38/p38 (n = 3). Compared to the control group, * represents *p* < 0.05, and ** represents *p* < 0.01; compared to the ET group, # indicates *p* < 0.05, and ## indicates *p* < 0.01; compared to the D-limonene interevent group, $$ indicates *p* < 0.01. Abbreviations: FCEO, finger citron essential oil; C, control group; ET, ethanol treatment group; FL, FCEO low-dose group; FM, FCEO medium-dose group; FH, FCEO high-dose group; LM, D-limonene group; Nrf2, nuclear factor erythroid 2-related factor 2; HO-1, heme oxygenase 1; NQO1, NADPH:quinone oxidoreductase 1; p-JNK, phosphor-c-Jun N-terminal kinase; p-ERK, phosphor-extracellular regulated protein kinase; p-p38, phosphor-p38 mitogen-activated protein kinase.

To elucidate the hepatoprotective mechanisms of FCEO, antioxidant enzymatic activity and redox-related protein expression were assessed. As shown in Figure 6, GSH levels significantly decreased in the ET group. After FCEO or D-limonene treatment, GSH levels had different degrees of retracement and were significantly different compared to the ET group (*F*_5, 30_ = 12.97) (Figure 6a). Also, there was a dose–effect relationship in the FCEO treatment groups. We also found that alcohol intake could downregulate liver tissue Nrf2 (*F*_5, 12_ = 23.09), HO-1 (*F*_5, 12_ = 2.75), and NQO1 (*F*_5, 12_ = 17.79) protein concentrations. After medium- or high-dose FCEO or D-limonene intervention, Nrf2, HO-1, and NQO1 protein expression increased. Notably, high-dose FCEO intervention induced significantly higher protein expression levels of Nrf2 and NQO1 compared to D-limonene intervention (Figure 6b–e).

Alcohol intake activated the MAPK pathway, characterized by an increase in the phosphorylation levels of JNK (*F*_5, 12_ = 3.383), ERK (*F*_5, 12_ = 3.66), and p38 (*F*_5, 12_ = 7.53) (Figure 6g–j). After the FCEO gavage intervention, the phosphorylation levels of JNK, ERK, and p38 lowered, while D-limonene treatment only significantly affected p38 phosphorylation levels. Also, a dose–effect relationship was found in the FCEO treatment groups. The above phenomena indicate that both FCEO and D-limonene could alleviate the adverse effects of alcohol intake on the redox equilibrium in ALD rats by regulating Nrf2 and the MAPK pathway, and FCEO had a better effect than D-limonene.

### 3.8. Effects of FCEO and D-Limonene Intervention on Inflammatory Factors and NF-κB/AMPK Pathway in ALD Rats

**Figure 7:**
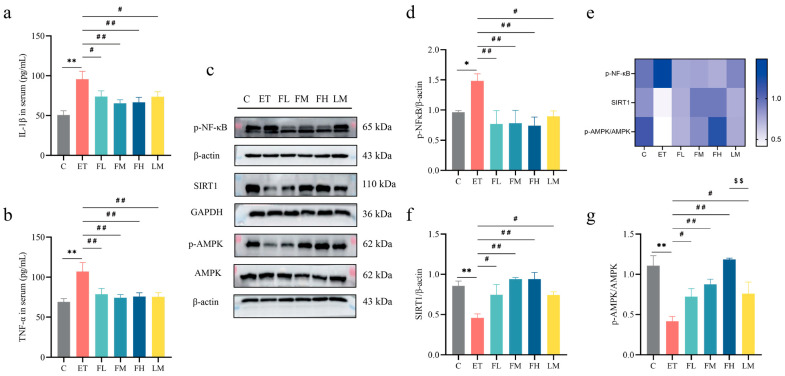
Effect of FCEO and/or D-limonene treatment on inflammatory factors and p-NF-κB/AMPK pathways in liver tissue of ALD rats: (**a**) IL-1β content in serum, (**b**) TNF-α content in serum (n = 6), (**c**) Western Blot strip map of p-NF-κB/AMPK pathway proteins, (**d**) p-NF-κB, (**e**) heat map corresponding to the content of proteins associated with the Nrf2 pathway, (**f**) SIRT/β-actin, and (**g**) p-AMPK/AMPK (n = 3). Compared to the control group, * represents *p* < 0.05, and ** represents *p* < 0.01; compared to the ET group, # indicates *p* < 0.05, and ## indicates *p* < 0.01; compared to the D-limonene interevent group, $$ indicates *p* < 0.01. *η*^2^ > 0.14; confidence intervals = 95%. Abbreviations: FCEO, finger citron essential oil; C, control group; ET, ethanol treatment group; FL, FCEO low-dose group; FM, FCEO medium-dose group; FH, FCEO high-dose group; IL-1β, interleukin-1beta; TNF-α, tumor necrosis factor-α; p-NF-κB, phosphor-nuclear factor-kappa B; SIRT1, sirtuin 1; p-AMPK, phosphor-AMP-activated protein kinase.

In the following part of the study, we evaluated the anti-inflammatory efficacy of FCEO in ALD rats and compared its effects with D-limonene through a comparative experiment. As shown in Figure 7a–d, alcohol intake caused a significant increase in IL-1β (*F*_5, 30_ = 4.79) and TNF-α (*F*_5, 30_ = 4.21) content in serum. Also, there was a significant increase in the phosphorylation levels of NF-κB in liver tissue, demonstrating that inflammation was present in the livers of the ALD rats. After FCEO or D-limonene intervention, serum IL-1β and TNF-α concentrations significantly decreased, and NF-κB phosphorylation levels significantly decreased (*F*_5, 12_ = 3.43). And there was no significant difference between the D-limonene intervention group and the FCEO intervention groups.

As shown in Figure 7e,f, the progression of inflammation in ALD was closely related to the AMPK signaling pathway. Hence, we also measured the expression of the key proteins in the AMPK pathway, SIRT1 and AMPK. Alcohol intake led to significant reductions in the expression of SIRT1 (*F*_5, 12_ = 6.22) and the phosphorylation level of AMPK (*F*_5, 12_ = 8.84). After the FCEO or D-limonene interventions, SIRT1 expression and AMPK phosphorylation levels in the liver significantly increased.

These findings suggest that both FCEO and D-limonene modulated the levels of proinflammatory factors, and the improvements might have been achieved through the NF-κB/AMPK pathway, thereby alleviating the inflammatory response in the livers of ALD rats. Furthermore, these results demonstrate that D-limonene is a principal active component for the inflammation-modulating effects of FCEO.

## 4. Discussion

In this study, we investigated the hepatoprotective effects of FCEO against alcohol-induced liver injury in rats, providing a comparative analysis of its efficacy relative to its primary constituent, D-limonene, as well as an exploration of the underlying mechanisms. The results demonstrated that both FCEO and D-limonene interventions attenuated alcohol-related hepatic histopathological alterations, including hepatocyte steatosis (irregular fatty vacuoles) and inflammatory infiltration. Additionally, they helped to downregulate the abnormal increase in serum transaminase levels triggered by alcohol. The hepatoprotective effects of these interventions are mechanistically linked to these compounds’ capacity to attenuate inflammatory cascades and counteract oxidative stress, achieved through the suppression of pro-inflammatory mediators. In comparison, the high-dose FCEO intervention was better than the low-dose and medium-dose interventions, and the medium and high-dose FCEO interventions were better than the D-limonene intervention in some respects.

Current research continues to demonstrate the therapeutic potential of Chinese herbal medicine and medicinal food homology substances in preventing alcohol liver disease. Conventional Western approaches predominantly employ nutritional interventions, including high-protein diets enriched with fatty acids and vitamin B/C supplementation [28]. Pharmacotherapies such as glucocorticoid, pentoxifylline, and metadoxine supplementation remain mainstream ALD treatments, yet their clinical utility is constrained by their adverse effects and single-target mechanisms [29]. In contrast, traditional Chinese medicine offers unique polypharmacological advantages through multi-component, multi-target mechanisms that synergistically modulate pathogenic pathways and provides superior safety profiles. These features not only address ALD pathology but also enhance systemic homeostasis. Puerarin, the main bioactive constituent of wild Gregory, has shown therapeutic efficacy in treating various different inflammatory diseases, including hepatitis, and been demonstrated to have hepatoprotective effects against ALD [30]. The polysaccharide derived from *Schisandra chinensis* has been shown to modulate gut microbiota composition in ALD murine models, which attenuates alcohol-induced hepatic inflammation, lipid accumulation, and oxidative stress [31]. Classified in traditional Chinese medicine, finger citron demonstrates Qi-regulating capacities essential for maintaining vital energy balance [32]. In recent years, with in-depth research on finger citron conducted both domestically and internationally, it was found that its essential oil could be used for stress reduction [33], and its juice could limit high-fat-diet-induced increases in liver weight in rats [34]. Limonene has been identified as the most abundant component in FCEO [35,36] and been shown to possess antioxidant [37] and hepatoprotective properties [25]. Therefore, we were curious about whether FCEO also has hepatoprotective properties and could be applied to alleviate alcoholic liver damage. We first demonstrated in vitro that FCEO modulated hepatic metabolic enzymes, providing a foundation for studying its hepatoprotective effects. Moreover, the novelty of our study is that we compared the hepatoprotective effects of FCEO and D-limonene in ALD model rats. This comparison not only allowed us to understand their hepatoprotective mechanisms but also helped us to determine whether the whole plant extracts or a monomer of FCEO would be more effective in preventing the progression of ALD.

Chronic alcohol consumption induces a pathological upregulation of CYP2E1, a cytochrome P450 oxidase mediating the oxidation of ethanol into acetaldehyde, during hepatic ethanol catabolism. An accumulation of large amounts of acetaldehyde will cause liver damage [38]. Studies have reported that CYP3A4 participates in the metabolism of approximately 50% of commonly prescribed medications [39]. Reduced enzymatic activities of CYP1A2 and CYP3A4 have been shown to attenuate liver injury in rats [40], and acute alcoholic injury in mice could be alleviated by inhibiting the expression of CYP2E1 [41]. Our findings revealed that FCEO treatment could downregulate the expression of the phase I metabolic enzymes CYP1A2, CYP2E1, and CYP3A4 in BRL-3A cells, and low doses of FCEO could increase the content of the Phase II metabolic enzyme GST. Collectively, these results suggest that the in vitro administration of FCEO has a modulatory influence on hepatic metabolic enzymes, potentially enhancing the hepatic metabolic capacity for drugs or exogenous toxins.

Continuous 28-day edible alcohol gavage led to the successful establishment of ALD models. Consequently, the bodyweight gain of the rats was slowed, falling to levels significantly lower than those of the control group and intervention groups, accompanied by a notable increase in the hepatic index. The histopathological examination of the livers of rats revealed irregular vacuoles, inflammation of hepatocytes (HE staining), and liver fattening (Oil Red O staining) in the ET group. Significant increases in ALT and AST activities were found in the ET group rats, and high levels of serum transaminase usually signify liver tissue damage. The negative effects of alcohol intake on the rat livers could be alleviated by different doses of FCEO or D-Limonene gavage treatment. Although the treatment did not help fully restore liver function to normal levels, inflammatory factor infiltration and lipid droplet accumulation in hepatocytes were reduced, and liver injury was alleviated.

Nrf2 plays a crucial role in attenuating oxidative stress, and activating the Nrf2 signaling pathway can alleviate ALD [42]. By activating the Nrf2 pathway, green tea extract can increase the levels of antioxidant enzymes, such as SOD, CAT, and GSH-Px, and restore autophagy in mouse livers, thereby ameliorating alcoholic liver injury [43]. This effect may involve the role of activated Nrf2 in maintaining lipid metabolism homeostasis. Moreover, the activation of Nrf2 by endogenous metabolites is also of great significance for modulating inflammatory responses [44]. In our study, FCEO and D-limonene were found to activate the Nrf2 signaling pathway, leading to a significant increase in GSH levels. The downstream target genes of Nrf2, such as HO-1 and NQO1, also play important roles in relieving alcoholic liver damage [45,46]. Under oxidative stress or other chemical stimuli, the nuclear translocation of Nrf2 involves its migration from cytoplasmic compartments to the nuclear domain, subsequently interacting with ARE motifs in gene-regulatory regions. This process initiates the transcriptional activation of phase II detoxifying enzymes, including HO-1 and GSH. HO-1 scavenges excess intracellular ROS and reduces oxidative damage, and NQO1 rapidly eliminates toxic substances and oxidative products from the body, thereby reducing damage to the liver. In our study, different dosages of FCEO and D-limonene upregulated the expression of the antioxidant proteins HO-1 and NQO1 and significantly increased GSH levels, thereby enhancing the rats’ antioxidant capacity and thus their ability to resist alcohol liver injury. This observation aligns with the mechanism proposed by Fang et al. [46]. Therefore, we speculated that alcohol consumption might disrupt redox homeostasis by inhibiting the expression of Nrf2, further suppressing the expression of HO-1 and NQO1, and exerting adverse effects on the antioxidant system. By activating Nrf2, FCEO and D-limonene increased HO-1 and NQO1 protein expression, which was beneficial in mitigating oxidative-stress-induced hepatic damage.

Long-term alcohol consumption triggers the release of endotoxins and inflammatory factors in hepatocytes, activating the NF-κB pathway. MAPK pathway activation exacerbates inflammatory–oxidative stress crosstalk during the pathogenesis of ALD [47]. When NF-κB-inducing kinase is stimulated to activate and promote the phosphorylated expression of IκB kinase, NF-κB is released from the IκB/NF-κB complex and migrates to the nucleus of the cell, accelerating the secretion and release of inflammatory factors, such as TNF-α, IL-1β, and IL-6, which contribute to hepatocellular necrosis [48]. Notably, inflammatory factor levels were abnormally elevated in the ET group, and the FCEO or D-limonene intervention helped to restore them to normal levels. This implies that restoring inflammatory factor levels using FCEO seems to be a feasible way to treat ALD. We also found that different dosages of FCEO or D-limonene inhibited the MAPK pathway. This inhibition may reduce the inflammatory cascade in the livers of ALD rats, promote hepatocyte proliferation, and reduce hepatocyte apoptosis, thereby effectively protecting the cells [49,50].

The SIRT1/AMPK pathway is well known for its ability to regulate inflammatory responses and metabolic disorders [51], especially in relation to an alcoholic liver injury [52]. Under homeostatic conditions, SIRT1 regulates the deacetylation of AMPK proteins, thereby regulating liver fat synthesis and catabolism [53]. Excessive alcohol metabolism can induce the conversion of large amounts of NAD+ to NADH, which inhibits the function of the SIRT1 protein, resulting in the acetylation of AMPK, promoting lipid generation and accumulation [54]. The activity of AMPK is inhibited during the process of ethanol metabolism, which in turn inhibits the release of peroxisome proliferator-activated receptor-α (PPAR-α), disrupts lipid metabolism, and inhibits antioxidant enzyme activities, resulting in the development of a fatty liver over time [55]. Promoting fatty acid oxidation and thus inhibiting lipid accumulation by increasing PPAR-α protein expression can improve the symptoms of alcoholic fatty liver disease [56]. In our study, interventions with FCEO and D-limonene alleviated the negative effects of alcohol treatment on SIRT1 and AMPK proteins, promoting lipid metabolism in hepatocytes and resulting in a decrease in the inflammatory response. Zhou et al. [57] and Zhuge et al. [58] have demonstrated that activating the SIRT1/AMPK pathway effectively ameliorates alcohol-induced liver injury. Hence, we believe that FCEO could alleviate alcoholic liver injury through a similar mechanism, and D-limonene is the main anti-inflammatory component. FCEO has exhibited dual regulatory capacity in attenuating alcohol-induced oxidative stress and inflammatory cascades in ALD rats, positioning it as a promising multi-target hepatoprotective candidate akin to established botanical agents such as puerarin and Schisandra chinensis polysaccharides.

In general, the complex phytochemical composition of plant extracts enables them to interact with multiple targets, thereby contributing in various ways to biological activity. This can be interpreted as the synergistic effect of multiple active molecules, which differs from the effects of individual components. Consequently, when investigating the biological activity of a novel plant extract, it is important to compare its biological effects with those of its monomeric components. This is especially crucial when the aim of research is to treat or alleviate the effects of a specific disease, such as the alcohol-induced liver disease discussed herein, wherein comparing the effects of FCEO and its monomers is imperative. By analyzing serum ALT, AST, TG, and TC levels alongside hepatic TG and TC levels, we demonstrated the regulatory effects of FCEO and D-limonene on metabolic enzymes and blood lipid metabolism in ALD rats. In the assessment of hepatic GSH and the MAPK/Nrf2 signaling pathways, we compared their redox-modulating capacities, while measurements of serum inflammatory cytokines (IL-1β, TNF-α) and NF-κB/AMPK pathway proteins explored their impacts on alcohol-induced inflammatory cascades. It is evident that D-limonene is the primary active component of FCEO. Nevertheless, in certain aspects, such as modulating the Nrf2 pathway, FCEO demonstrated more pronounced effects. This phenomenon may be due to the contribution of additional components, including α-pinene and γ-terpinene, phytochemicals documented for their ability to scavenge oxidative free radicals [59,60]. Since this study focused on comparing FCEO with its predominant constituent, the potential contributory roles of minor components such as γ-Terpinene and o-Cymene in the observed hepatoprotective effects cannot be fully excluded. To conclusively establish whether the therapeutic efficacy of FCEO stems from its holistic mixture rather than its individual constituents, future investigations should incorporate systematic comparative studies including o-Cymene and γ-Terpinene comparison groups. Furthermore, mechanistic validations employing inhibitors (e.g., ML385 for Nrf2 inhibition) and siRNA-mediated gene knockdown targeting key oxidative-inflammatory crosstalk mediators would provide critical insights into the pathway-specific modulation exerted by all the components of FCEO. Metabolomics could also be employed in the future to comprehensively characterize the bioactive metabolites and their temporal dynamics during the biotransformation of FCEO in rat models, with a particular focus on the specific metabolite clusters associated with alcohol liver injury mitigation.

## 5. Conclusions

Our in vitro analyses demonstrated the capacity of FCEO to modulate phase I and II metabolic enzymes, while our in vivo investigations revealed that both FCEO and its main component, D-limonene, attenuated alcohol-induced hepatic injury through modulation of oxidative–inflammatory crosstalk. Specifically, FCEO and D-limonene played a regulatory role in oxidative-stress- and inflammation-related pathways such as the MAPK/Nrf2 and NF-κB/AMPK pathways. D-limonene may greatly contribute to the hepatoprotective effects of FCEO, while whole-component FCEO is more effective. Therefore, in the context of multi-target pharmacological strategies for ALD, FCEO holds great potential for development and application.

## Figures and Tables

**Table 1:** Treatments for each experimental group.

Groups	Gavage Sessions	
	9:00 a.m.	2:00 p.m.
C	corn oil	saline
ET	corn oil	edible alcohol
FL	100 mg/kg of FCEO	edible alcohol
FM	200 mg/kg of FCEO	edible alcohol
FH	400 mg/kg of FCEO	edible alcohol
LM	116 mg/kg of D-limonene	edible alcohol

The intervention dose in the LM group was calculated according to the D-limonene content corresponding to the FCEO volume administered to the FM group. Abbreviations: FCEO, finger citron essential oil; C, control group; ET, ethanol treatment group; FL, FCEO low-dose group; FM, FCEO medium-dose group; FH, FCEO high-dose group; LM, D-limonene group.

**Table 2:** Volatile components of finger citron essential oil.

No.	RT (min)	Compound	CAS	Relative Percentage (%)
1	13.17	Bicyclo[3.1.0]hex-2-ene, 4-methyl-1-(1-methylethyl)-	28634-89-1	1.12
2	13.50	α-Pinene	80-56-8	2.73
3	14.61	α-Phellandrene	99-83-2	0.39
4	14.82	β-Myrcene	123-35-3	1.28
5	14.87	β-Pinene	127-91-3	2.21
6	16.18	o-Cymene	527-84-4	10.66
7	16.36	D-limonene	5989-27-5	57.97
8	16.66	3-Carene	13466-78-9	1.32
9	17.31	γ-Terpinene	99-85-4	21.54
10	18.42	Cyclohexene, 1-methyl-4-(1-methylethylidene)-	586-62-9	0.76

RT: retention time.

## Data Availability

Data are contained within the article.

## Supplementary Materials

The following supporting information can be downloaded at https://www.mdpi.com/article/10.3390/nu17071255/s1. Figure S1: Essential oil components of Finger citron.

## Abbreviations

The following abbreviations are used in this manuscript:

FCEO	Finger citron essential oil
ALD	Alcohol-induced liver disease
TG	Triglyceride
TC	Total cholesterol
GSH	Glutathione
BRL-3A	Buffalo rat liver-3A
GC-MS	Gas chromatograph-mass spectrometer
RT	Retention time
BSA	BSA
PBS	Phosphate buffered saline
TBST	TBST
DMSO	Dimethyl sulfoxide
PMSF	Phenylmethanesulfonyl fluoride
PVDF	Polyvinylidene difluoride
ALT	Alanine aminotransferase
AST	Aspartate aminotransferase
UGT	Uridine diphosphate-galactose transporter 1
Nrf2	Nuclear factor erythroid 2-related factor 2
HO-1	Heme oxygenase 1
NQO1	NADPH:quinone oxidoreductase 1
NF-κB	Nuclear factor-kappa B
IκB	Inhibitor of NF-κB
IL-1β	Interleukin-1beta
MAPK	Mitogen-activated protein kinase
JNK	c-Jun N-terminal kinase
ERK	Extracellular regulated protein kinase
p38	p38 Mitogen-activated protein kinase
AMPK	AMP-activated protein kinase
SIRT1	Sirtuin 1
CYP	Cytochrome P450 proteins
GST	Glutathione S-transferase
TNF-α	Tumor necrosis factor-α
NF-κB	Nuclear factor-kappa B
