# Supplementary material for: Role in Preventing Alcoholic Liver Disease Progression: A Comparative Study of Whole-Component Finger Citron Essential Oil and Its Major Component D-Limonene
Source: Nutrients. 2025 Apr 3;17(7):1255. doi: 10.3390/nu17071255 (PMC11990129; doi:10.3390/nu17071255)
Supplement: Supplementary file 1 [file nutrients-17-01255-s001.zip › nutrients-3539311-supplementary.pdf]

Article

Role in Preventing Alcoholic Liver Disease Progression: A Comparative Study of Whole-Component Finger Citron Essential Oil and Its Major Component D-limonene

Jingxin Chen [1,2,†], Genghua Ou [1,2,†], Wenting Gu [1,2], Jian Shi [1,2], Ruiying Lyu [1,2], Xueping Wu [1,2], Junming Wang [1,2], and Chunhong Liu [1,2,*]

1 College of Food Science, South China Agricultural University, Guangzhou 510642, China; e-mail@e-mail.com
2 Guangdong Provincial Key Laboratory of Food Quality and Safety, Guangzhou 510642, China; e-mail@e-mail.com
* Correspondence: liuch@scau.edu.cn; Tel.: (020) 85283448
† Authors contributed equally to this paper.

Supplementary figure

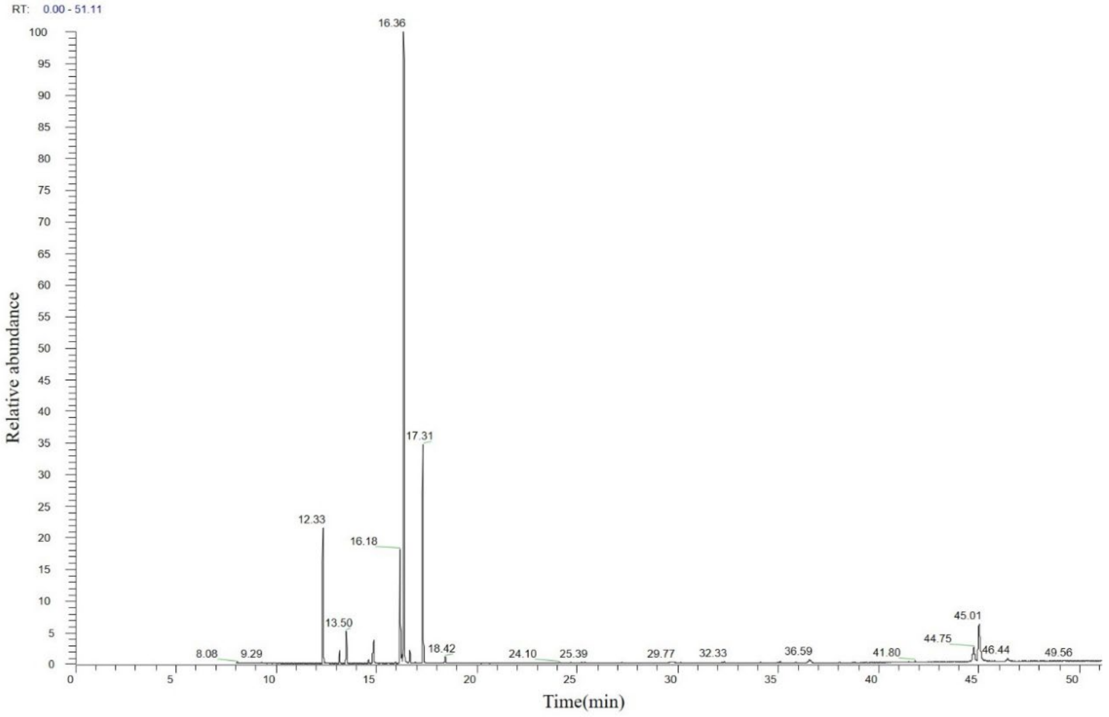

Supplementary Figure 1. Essential oil components of Finger citron.
